# Analysis of funding landscape for health policy and systems research in the Eastern Mediterranean Region: A scoping review of the literature over the past decade

**DOI:** 10.1186/s12961-024-01161-3

**Published:** 2024-06-24

**Authors:** Racha Fadlallah, Fadi El-Jardali, Nesrin Chidiac, Najla Daher, Aya Harb

**Affiliations:** 1 Department of Health Management and Policy, Faculty of Health Sciences, American University of Beirut, Beirut, Lebanon; 2 Health Systems Global Society, London, UK; 3 Knowledge to Policy (K2P) Center, American University of Beirut, Beirut, Lebanon; 4 Department of Health Research Methods, Evidence, and Impact (HEI), McMaster University, Ottawa, ON Canada

**Keywords:** Health policy and systems research, Funding, Funding sources, Eastern Mediterranean Region, Scoping review

## Abstract

**Background:**

Health policy and systems research (HPSR) can strengthen health systems and improve population health outcomes. In the Eastern Mediterranean Region (EMR), there is limited recognition of the importance of HPSR and funding remains the main challenge. This study seeks to: (1) assess the reporting of funding in HPSR papers published between 2010 and 2022 in the EMR, (2) examine the source of funding in the published HPSR papers in the EMR and (3) explore variables influencing funding sources, including any difference in funding sources for coronavirus disease 2019 (COVID-19)-related articles.

**Methods:**

We conducted a rapid scoping review of HPSR papers published between 2010 and 2022 (inclusively) in the EMR, addressing the following areas: reporting of funding in HPSR papers, source of funding in the published HPSR papers, authors’ affiliations and country of focus. We followed the Joanna Briggs Institute (JBI) guidelines for conducting scoping reviews.

We also conducted univariate and bivariate analyses for all variables at 0.05 significance level.

**Results:**

Of 10,797 articles screened, 3408 were included (of which 9.3% were COVID-19-related). More than half of the included articles originated from three EMR countries: Iran (*n* = 1018, 29.9%), the Kingdom of Saudi Arabia (*n* = 595, 17.5%) and Pakistan (*n* = 360, 10.6%). Approximately 30% of the included articles did not report any details on study funding. Among articles that reported funding (*n* = 1346, 39.5%), analysis of funding sources across all country income groups revealed that the most prominent source was national (55.4%), followed by international (41.7%) and lastly regional sources (3%). Among the national funding sources, universities accounted for 76.8%, while governments accounted for 14.9%. Further analysis of funding sources by country income group showed that, in low-income and lower-middle-income countries, all or the majority of funding came from international sources, while in high-income and upper-middle-income countries, national funding sources, mainly universities, were the primary sources of funding. The majority of funded articles’ first authors were affiliated with academia/university, while a minority were affiliated with government, healthcare organizations or intergovernmental organizations. We identified the following characteristics to be significantly associated with the funding source: country income level, the focus of HPSR articles (within the EMR only, or extending beyond the EMR as part of international research consortia), and the first author’s affiliation. Similar funding patterns were observed for COVID-19-related HPSR articles, with national funding sources (78.95%), mainly universities, comprising the main source of funding. In contrast, international funding sources decreased to 15.8%.

**Conclusion:**

This is the first study to address the reporting of funding and funding sources in published HPSR articles in the EMR. Approximately 30% of HPSR articles did not report on the funding source. Study findings revealed heavy reliance on universities and international funding sources with minimal role of national governments and regional entities in funding HPSR articles in the EMR. We provide implications for policy and practice to enhance the profile of HPSR in the region.

**Supplementary Information:**

The online version contains supplementary material available at 10.1186/s12961-024-01161-3.

## Introduction

The coronavirus disease 2019 (COVID-19) pandemic has demonstrated how vulnerabilities in health systems can have profound implications for health, economic progress, trust in governments and social cohesion [1–3]. Strengthening the capacity of health systems to respond swiftly and effectively has become a priority for governments worldwide as they emerge from the pandemic [4]. Health policy and systems research (HPSR) can provide context-relevant knowledge to strengthen health systems and improve population health outcomes [5–7]. In spite of international calls to increase investments in HPSR, studies suggest that less than 2% of global health funding is being spent on health systems strengthening and HPSR. This lack of adequate funding is especially an issue in low- and middle-income countries (LMIC), where funding remains largely dependent on external sources [8]. This is further challenged by the near invisibility of domestic funding flow for HPSR on a national level [9].

In the Eastern Mediterranean Region (EMR), while much of the policy priorities are related to health systems, there is poor recognition of the importance of HPSR [10–12], and funding limitations remain the main challenge facing HPSR in the region [11, 13]. Given that the advancement of HPSR is greatly dependent on the availability of adequate and reliable funding [14, 15], it would be important to gain a better understanding of HPSR funding in the EMR. Previous studies have assessed funding for HPSR at the level of national government and from international donor perspectives [11, 14]. Rabbat et al. assessed funding for HSPR in the EMR on a national level and found that none of the EMR countries have explicit national funding or a budget line for HPSR [11]. Grepin et al. analysed donor funding for HPSR in LMICs, including EMR, and found that such funding is heavily concentrated, with more than 93% coming from just 10 donors, and only represents approximately 2% of all donor funding for health and population projects. Moreover, countries in the sub-Saharan African region were the major recipients of HPSR funding, while countries in the EMR were the least recipients of such funding [14].

The current study adds to existing literature by analysing the sources of funding for published HPSR studies in countries of the EMR. Analysis of HPSR publications in countries from the region can be used to monitor progress and trends in the production of policy-relevant research and is a core requirement for strengthening health research systems to generate and use knowledge to improve health systems [8, 16]. The specific objectives are to: (1) assess the reporting of funding in HPSR papers published between 2010 and 2022 in the EMR, (2) examine the source of funding in the published HPSR papers in the EMR and (3) explore variables influencing funding sources, including any difference in funding sources for COVID-19-related articles. Findings will enable a better understanding of the HPSR funding landscape in the EMR.

## Methods

We conducted a rapid scoping review of HPSR papers published between 2010 and 2022 in the EMR, addressing the following broad areas: reporting of funding in HPSR papers, source of funding in the published HPSR papers, authors’ affiliations and country of focus. Scoping reviews are an ideal tool to convey the breadth and depth of a body of literature on a given topic and give clear indication of the volume of literature and studies available as well as an overview of its focus [17]. We followed standard methodology and the Preferred Reporting Items for Systematic Reviews and Meta-Analyses extension for Scoping Reviews (PRISMA-ScR) guidelines for reporting scoping reviews (Supplementary file 1) [18].

### Eligibility criteria

Study design: All study designs were included except for letters, correspondence, commentaries, dissertations, technical papers, handbooks, protocols and editorials. We restricted the search date to studies published in the last decade (that is, 2010–2022, inclusive).

Setting: Eastern Mediterranean Region. We included all countries established within the WHO’s Eastern Mediterranean Regional Office, namely Afghanistan, Bahrain, Djibouti, Egypt, the Islamic Republic of Iran, Iraq, Jordan, Kuwait, Lebanon, Libya, Morocco, Oman, Pakistan, the Occupied Palestinian Territories, Qatar, Saudi Arabia, Somalia, South Sudan, Sudan, Syrian Arab Republic, Tunisia, the United Arab Emirates and Yemen.

Population: We did not limit the search to any specific type of population.

Dimensions of interest: We considered studies to be eligible if they met the criteria of health systems topics developed by the McMaster Health Forum, including governance, financial and delivery arrangements, and implementation strategies. The selected coding framework has been previously implemented for coding health policy and systems topics in countries from the region [19, 20].

We did not restrict the search to any language.

### Literature search

We searched the following electronic databases in December 2022: PubMed, Web of Science, The Index Medicus for the Eastern Mediterranean Region (IMEMR) and Google Scholar. We used both index terms and free text words for the following two concepts (and their variations): (1) EMR countries and (2) HPSR. The search strategy was validated with the guidance of an information specialist. For the Web of Science, we limited the search to 72 journals listed under the “Health Policy and Services” (HPS) category in Web of Science between 2010 and 2022. A similar approach was previously adopted in a study examining the reporting of funding in HPSR [19]. We also screened the reference lists of all included articles.

### Study selection and data extraction

Prior to proceeding with the selection process, we conducted a calibration exercise to enhance validity of the selection process. Two reviewers used the above eligibility criteria to screen the identified citations for potential eligibility. Half of the included studies were screened in duplicate and independently by teams of two reviewers, while the remaining were screened independently by each reviewer. We obtained the full text for citations judged as potentially eligible by at least one of the reviewers. To enhance validity of the process, all excluded studies were validated by a senior reviewer (who is the senior author). Any disagreement was resolved by discussion, and when needed, with the help of a third reviewer.

We developed a data extraction form. Prior to proceeding with the selection process, we conducted a calibration exercise to enhance validity of the selection process. Each of four reviewers independently abstracted data from a subset of articles assigned to them (collectively covering the full dataset), using a standardized and pilot-tested form. Throughout the process, all team members were consulted to validate coding decisions. Any disagreement was resolved by discussion, and when needed, with the help of a third reviewer. We revisited and considered data in the context of any newly emergent decision. Additionally, the coding sections related to reported source of funding and first authors’ affiliations were independently validated by a second reviewer.

The following information was abstracted from all included studies:

- Study ID (author last name, title of study).
- Date of publication.
- Type of article.
- Country subject of the paper: This refers to the geographical scope of the article, specifically the country where the research was conducted. Articles encompassing more than one country were categorized into two distinct groups: those focusing solely on the EMR (referred to as “more than one within the region”), and those extending beyond the EMR (referred to as “more than one beyond the region”) – maintaining a distinction from individual country analyses.
- Country income group classification (first country) as per World Bank classification data for 2021–2022.
- Reported affiliation(s) by the first author.
- Reported affiliation(s) by the corresponding author.
- Country of the institution to which the first author is affiliated.
- Country of the institution to which the corresponding author is affiliated.
- Reporting of study funding (not reported, reported as funded or reported as not funded).
- Reported source(s) of funding.
- Whether the study was COVID-19-related.

### Statistical analysis

We conducted univariate and bivariate analyses for all variables collected for the included papers using IBM Statistical Package for Social Sciences (SPSS) Statistics v.25. We used the chi-square test at 0.05 significance level to compare categorical data and investigate the associations between reporting of funding by papers, type of funding and income groups. We also used the chi-square test to examine whether significant associations exist between funding sources and the following variables: country income level, country focus of the HPSR articles (that is, within the EMR only, or beyond the EMR as part of international research consortia), the first author’s country of affiliation and COVID-19-related studies.

## Results

### Characteristics of included articles

**Fig. 1:**
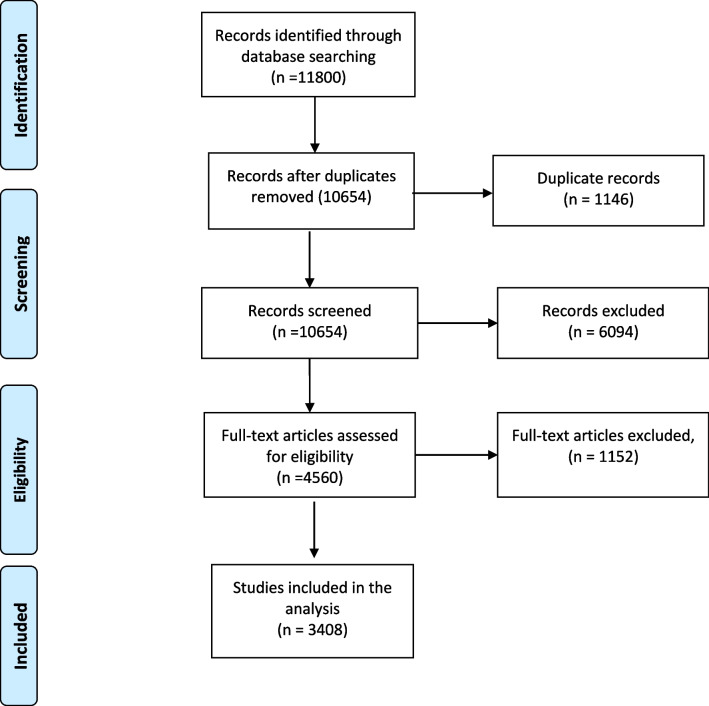
PRISMA flowchart

Figure 1 shows the PRISMA flowchart for study selection. Of 10,797 articles screened, 3408 were included.

**Fig. 2:**
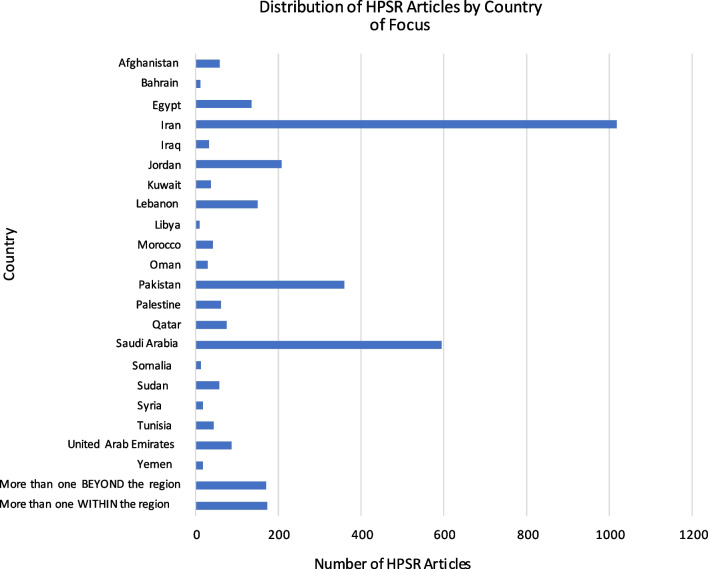
Distribution of HPSR articles by country of focus (*N* = 3408)

Figure 2 illustrates the distribution of HPSR articles by country focus. The top country focus was Iran (*n* = 1018, 29.9%), followed by Saudi Arabia (*n* = 595, 17.5%), Pakistan (*n* = 360, 10.6%), Jordan (*n* = 208, 6.1%) and Lebanon (*n* = 150, 4.4%). Notably, more than half of the included articles originated from only three EMR countries: Iran, the Kingdom of Saudi Arabia (KSA) and Pakistan. Countries where the fewest number of articles were conducted were Libya (*n* = 10, 0.3%), Bahrain (*n* = 12, 0.4%), Somalia (*n* = 13, 0.4%), Syria (*n* = 18, 0.5%), and Yemen (*n* = 18, 0.5%). No articles were found focusing on Djibouti only. Approximately 10% of the included articles were conducted in more than one EMR country. Of these, 5% focused on multiple countries within the EMR, while an additional 5% included at least one country beyond the EMR region (along with at least one within the EMR).

COVID-19-related articles accounted for 9.3% of the total HPSR studies published. Of these, the Kingdom of Saudi Arabia produced the highest number of publications (*n* = 93, 29.3%), followed by Iran (*n* = 68, 21.5%).

**Fig. 3:**
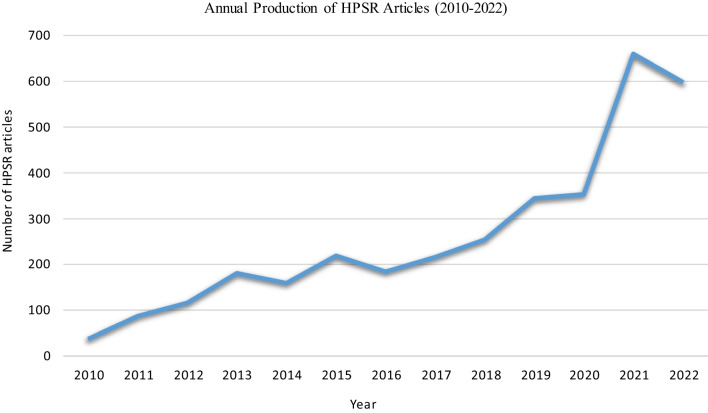
Annual production of HPSR articles from 2010 to 2022

Figure 3 illustrates the increase in the production of HPSR articles in the EMR from 2010 to 2022. The number of HPSR articles in 2010 was approximately 37, increasing to 598 by 2022. Furthermore, HPSR articles nearly doubled after 2020.

### Reporting of funding and funding sources for HPSR articles across country income groups

Table 1 presents the reporting of funding and sources of funding in the 3408 HPSR articles retrieved. Approximately 40% (*n* = 1346) of the articles reported being funded, while 29% (or 1001) did not report any details on study funding. It is worth noting that, while the number of funded articles is 1346, when taking into account all funding sources within an article, the total number of funding sources increases to 1635.

**Table 1:** Reporting of funding and funding sources for HPSR articles conducted in the EMR

Characteristics	*N* (%)
Study funding	*N* = *3408*
Not reported	1001 (29.4%)
Reported as funded	1346 (39.5%)
Reported as not funded	1061 (31.1%)

Sources of funding	n^*^ = *1635*
National funding sources^**^ Universities (76.8%) Government (14.9%) Health agencies (3.6%) Local charitable foundations/NGOs (3.2%) Pharmaceutical industry (1.4%) Other (0.12%)	905 (55.4%)
Regional funding sources^**^ Universities (53%) NGOs (17.7%) Government (13.3%) Health agencies (13.3%) Pharmaceutical industry (2.2%)	45 (2.8%)
International funding sources^**^ Governments (40.3%) Intergovernmental organizations (for example, UN agencies, World Bank, WHO) and NGOs (38.7%) Universities (10.8%) Pharmaceutical industry (5.86%) Health agencies (4.25%)	682 (41.7%)
Not clear	2 (0.1%)

First authors’ affiliations of funded articles	*N* = *1346*
Government	51 (3.8%)
Healthcare organizations	59 (4.4%)
Intergovernmental (WHO, UN, World Bank) organizations	25 (1.9%)
Not-for-profit organization	41 (3%)
Private academia/university	204 (15.2%)
Public academia/university	959 (71.2%)
Private for-profit	6 (0.4%)
Not reported	1 (0.1%)

Distribution of funded articles by income group^***^	*n* = *1346*
High-income country	272 (20.2%)
Upper-middle-income country	471 (35%)
Lower-middle-income country	453 (33.7%)
Low-income country	109 (8.1%)
More than one	41 (3%)

UN, United Nations; NGO, non-governmental organization.

^*^This section includes all the funding sources; even if one study has multiple funding sources, all of them are counted, and not only the main funding source

^**^With the exclusion of articles conducted in Iran, the distribution of funding sources becomes as follows: national (*n* = 336, 40.2%;), regional (*n* = 40, 4.8%), international (*n* = 457, 54.7%) and not clear (*n* = 2, 0.2%)

^***^The distribution is based on World Bank country classifications by income level for 2021–2022

Among the 1346 funded articles (with 1635 funding sources) in the EMR, analysis of funding sources across all country income groups revealed that the most common source was national (*n* = 905, 55.4%) followed by international (682, 41.7%) and lastly regional sources (41, 3%). Among the national funding sources, universities accounted for 76.8%, while governments accounted for 14.9% of the sources. Further analysis of funding sources by country income group showed that, in low-income and lower-middle-income countries (as classified by the World Bank at the time of data collection), all or the majority of funding came from international sources, while in high-income and upper-middle-income countries, national funding sources, mainly universities, were the primary sources. The majority of funded articles’ first authors were affiliated with academia/university distributed into public university (*n* = 959, 71.2%) and private (*n* = 204, 15.2%) while a minority were affiliated with government, private for-profit or intergovernmental organizations.

Of the COVID-19-related HPSR articles, 95 (or 30%) were funded, while 69 (or 21.8%) did not report any detail on the study funding. The most notable funding source was national (75, 78.95%), mainly universities (61.05%), with governments contributing to 10.53% of funding. International funding sources accounted for 15 (or 15.79%) of publications. Regarding the first authors’ affiliations of funded COVID-19-related articles, almost all authors are from the EMR and affiliated to public academic universities (80%).

### Source of funding and first-author affiliation for HPSR articles, by EMR country (2010–2020)

A breakdown of the source of funding by country is provided in Table 3.

All of the articles conducted in Somalia, Syria and Yemen and the majority of articles conducted in Afghanistan (76.27%), Egypt (62.39%), Iraq (66.5%), Lebanon (70.5%), Morocco (82.54%), Pakistan (80%), Palestine (95.8%), Sudan (80.6%) and Tunisia (89%) were funded by international sources. Articles conducted in the remaining EMR countries were largely funded by national sources; among those conducted in Iran (92.96%), Jordan (55.5%), the KSA (89%), Qatar (99.8%) and the United Arab Emirates (UAE; 68.2%), the majority were funded by universities, whereas in Libya (100%) and Kuwait (75%), the government was the main source of funder. Regarding Oman (46%), funding was equally distributed between national and international sources.

Concerning the first author’s affiliations, the majority of articles conducted in Egypt (71.1%), Iran (95.3%), Jordan (73.8%), the KSA (79.3%), Kuwait (70%), Morocco (63.2%), Oman (55.6%), Palestine (83.3%), Qatar (56.3%), Tunisia (66.7%), Somalia (100%), Syria (57.1%), the UAE (58.3%), Iraq (40%), and Yemen (100%) were affiliated with public academic institutions. In Lebanon (65.3%), Sudan (32%), and Pakistan (41.1%), the majority of the papers’ first authors were affiliated with private academia/university. In Afghanistan (27.8%), the papers’ first authors were equally affiliated with private university/academia and not-for-profit organizations. In Libya (100%), the first author of the only funded study was affiliated with intergovernmental organizations.

Across all income groups, the majority of the papers’ first authors were affiliated with academia/university. A minority of papers’ first authors was affiliated with private for-profit and intergovernmental organizations (Table 2).

**Table 2:** Source of funding and first-author affiliation for HPSR studies by EMR country

	Number of articles (Percentage out of the total articles, %) *N* = 3408	Funded articles (percentage out of the funded articles in the country, %) *N* = 1346	First-author affiliation of funded articles (*N*, percentage out of total funded articles for the country) *N* = 1346	Source of funding^*^ (*N*, percentage out of total funded articles for the country) *N* = 1632^*^
**High-income countries**				
Bahrain	12 (0.4%)	0 (0%)	• Not applicable; no studies funded	• Not applicable; no studies funded
Kuwait	37 (1.1%)	10 (0.7%)	• Government (2, 20%) • Healthcare organizations (1,10%) • Public academic/university (7, 70%)	• **National funding sources (15, 93.75%)** - NGOs (1, 6.25%) - Government (12, 75%) - Health agencies (1, 6.25%) - University/academia (1, 6.25%) • **Regional funding sources (0, 0%)** • **International funding sources (1, 6.25%)** - Government (1, 6.25%)
Oman	30 (0.9%)	9 (0.7%)	• Government (2, 22.2%) • Private academic/university (2, 22.2%) • Public academic/university (5, 55.6%)	• **National funding sources (6, 46%)** - Government (3, 23%) - University/academia (3, 23%) • **Regional funding sources (1, 8%)** - NGOs (1, 8%) • **International funding sources (6, 46%)** - NGOs (3, 23%) - Government (3, 23%)
Qatar	75 (2.2%)	32 (2.4%)	• Government (1, 3.1%) • Healthcare organizations (9, 28.1%) • Private academic/university (4, 12.5%) • Public academic/university (18, 56.3%)	• **National funding sources (34, 99.8%)** - NGOs (7, 20.5%) - Government (6, 17.6%) - Health agencies (8, 23.5%) - University/academia (13, 38.2%) • **Regional funding sources (0, 0%)** • **International funding sources (0, 0%)**
Saudi Arabia	595 (17.5%)	168 (12.5%)	• Government (9, 5.4%) • Healthcare organizations (10, 6%) • Intergovernmental (1, 0.6%) • Not-for-profit (1, 0.6%) • Private academic/university (13, 7.7%) • Public academic/university (133, 79.3%) • Private for-profit (1, 0.6%)	• **National funding sources (166, 89%)** - NGOs (3, 1.6%) - Government (18, 9.6%) - Health agencies (6, 3.2%) - University/academia (129, 69.3%) - Pharmaceutical industry (10, 5.3%) • **Regional funding sources (1, 0.5%)** - University/academia (1, 0.5%) • **International funding sources (18, 10.1%)** - NGOs (8, 4.3%) - Government (1, 0.5%) - University/academia (2, 1%) - Pharmaceutical industry (8, 4.3%)
United Arab Emirates	87 (2.6%)	20 (1.5%)	• Government (2, 16.7%) • Private academic/university (3, 25.0%) • Public academic/university (7, 58.3%)	• **National funding sources (15, 68.2%)** - Government (5, 22.7%) - Health agencies (1, 4.5%) - University/academia (9, 41%) • **Regional funding sources (2, 9%)** - University/academia (2, 9%) • **International funding sources (5, 22.6%)** - NGOs (2, 9%) - Pharmaceutical industry (3, 13.6%)
**Upper-middle-income countries**				
Iraq	33 (1%)	10 (0.7%)	• Healthcare organizations (1, 10%) • Intergovernmental (1, 10%) • Not-for-profit (1, 10%) • Private academic/university (3, 30%) • Public academic/university (4, 40%)	• **National funding sources (3, 25%)** - Government (3, 25%) • **Regional funding sources (1, 8.3%)** - NGOs (1, 8.3%) • **International funding sources (8, 66.5%)** - NGOs (5, 41.6%) - Government (1, 8.3%) - University/academia (1, 8.3%) - Pharmaceutical industry (1, 8.3%)
Iran^**^	1018 (29.9%)	511 (37.9%)	• Government (4, 0.8%) • Healthcare organizations (4, 0.8%) • Intergovernmental (2, 0.4%) • Not-for-profit (1, 0.2%) • Private academic/university (13, 2.5%) • Public academic/university (487, 95.3%)	• **National funding sources (518, 92.96%)** - NGOs (10, 1.79%) - Government (50, 9%) - Health agencies (8, 1.4%) - University/academia (449, 80.6%) - Pharmaceutical industry (1, 0.17%) • **Regional funding sources (1, 0.17%)** - Government (1, 0.17%) • **International funding sources (38, 6.72%)** - NGOs (18, 3.23%) - Government (6, 1%) - Health agencies (4, 0.71%) - University/academia (8, 1.43%) - Pharmaceutical industry (2, 0.35%)
Jordan	208 (6.1%)	80 (5.9%)	• Healthcare organizations (3, 3.8%) • Intergovernmental (1, 1.3%) • Private academic/university (17, 21.3%) • Public academic/university (59, 73.8%)	• **National funding sources (50, 55.5%)** - Government (1, 1.1%) - Health agencies (1, 1.1%) - University/academia (47, 52.2%) - Pharmaceutical industry (1, 1.1%) • **Regional funding sources (2, 2.2%)** - Health agencies (2, 2.2%) • **International funding sources (38, 42%)** - NGOs (12, 13.3%) - Government (15, 16.6%) - Health agencies (1, 1.1%) - University/academia (6, 6.6%) - Pharmaceutical industry (4, 4.4%) - Others
Libya	10 (0.3%)	1 (0.1%)	• Intergovernmental (WHO, UN, World Bank; 1, 100.0%)	• **National funding sources (1, 100%)** - Government (1, 100%) • **Regional funding sources (0, 0%)** • **International funding sources (0, 0%)**
**Lower-middle-income countries**				
Djibouti	0	0	–	–
Egypt	135 (4%)	45 (3.3%)	• Government (2, 4.4%) • Healthcare organizations (2, 4.4%) • Intergovernmental (WHO, UN, World Bank; 1, 2.2%) • Not-for-profit organization (1, 2.2%) • Private academic/university (5, 11.1%) • Public academic/university (32, 71.1%) • Private for-profit (2, 4.4%)	• **National funding sources (17, 30.34%)** - Government (10, 17.85%) - Health agencies (2, 3.57%) - University/academia (4, 7.14%) - Pharmaceutical industry (1, 1.78%) • **Regional funding sources (4, 7.14%)** - NGOs (2, 3.57%) - University/academia (2, 3.57%) • **International funding sources (35, 62.39%)** - NGOs (8, 14.2%) - Government (20, 35.7%) - University/academia (4, 7.14%) - Pharmaceutical industry (3, 5.35%)
Lebanon^**^	150 (4.4%)	49 (3.6%)	• Government (4, 8.2%) • Healthcare organizations (1, 2%) • Intergovernmental (1, 2%) • Not-for-profit organization (2, 4.1%) • Private academic/university (32, 65.3%) • Public academic/university (9, 18.4%)	• **National funding sources (17, 26%)** - NGOs (2, 3%) - Government (3, 4.6%) - University/academia (12, 18.4%) • **Regional funding sources (2, 3%)** - Government (1, 1.5%) - University/academia (1, 1.5%) • **International funding sources (46, 70.5%)** - NGOs (28, 43%) - Government (14, 21.5%) - Health agencies (2, 3%) - University/academia (2, 3%)
Morocco	42 (1.2%)	19 (1.4%)	• Government (1, 5.3%) • Intergovernmental (WHO, UN, World Bank; 2, 10.5%) • Not-for-profit (1, 5.3%) • Private academic university (3, 15.8%) • Public academic/university (12, 63.2%)	• **National funding sources (1, 4.34%)** - University/academia (1, 4.34%) • **Regional funding sources (3, 13.04%)** - Government (2, 8.7%) - University/academia (1, 4.34%) • **International funding sources (19, 82.54%)** - NGOs (11, 47.8%) - Government (3, 13%) - Health agencies (2, 8.7%) - University/academia (2, 8.7%) - Pharmaceutical industry (1, 4.34%)
Palestine	62 (1.8%)	18 (1.3%)	• Healthcare organizations (1, 5.6%) • Intergovernmental (WHO, UN, World Bank; 1, 5.6%) • Public academic/university (15, 83.3%) • Private for-profit (1, 5.6%)	• **National funding sources (1, 4.2%)** - University/academia (1, 4.2%) • **Regional funding sources (0, 0%)** • **International funding sources (23, 95.8%)** - NGOs (6, 25%) - Government (14, 58.3%) - Health agencies (3, 12.5%)
Pakistan	360 (10.6%)	124 (9.2%)	• Government (10, 8.1%) • Health agencies (for example, healthcare organization; 8, 6.5%) • Intergovernmental (WHO, UN, World Bank; 1, 0.8%) • Not-for-profit organization (11, 8.9%) • Private academic/university (51, 41.1%) • Public academic/university (41, 33.1%) • Not reported (1, 0.8%) • Private for-profit (1, 0.8%)	• **National funding sources (33, 18%)** - NGOs (4, 2.2%) - Government (7, 3.9%) - Health agencies (6, 3.3%) - University/academia (16, 8.8%) • **Regional funding sources (3, 2%)** - University/academia (3, 1.6%) • **International funding sources (145, 80%)** - NGOs (51, 28.1%) - Government (70, 38.6%) - Health agencies (7, 3.9%) - University/academia (17, 9.39%)
Tunisia	44 (1.3%)	9 (0.7%)	• Healthcare organizations (3, 33.3%) • Public academic/university (6, 66.7%)	• **National funding sources (0, 0%)** • **Regional funding sources (1, 11%)** - University/academia (1, 11.1%) • **International funding sources (8, 89%)** - NGOs (4, 44.4%) - Government (2, 22.2%) - University/academia (1, 11.1%) - Pharmaceutical industry (1, 11.1%)
**Low-income countries**				
Afghanistan	59 (1.7%)	36 (2.7%)	• Government (4, 11.1%) • Healthcare organizations (3, 8.3%) • Not-for-profit organization (10, 27.8%) • Private academic/university (10, 27.8%) • Public academic/university (9, 25%)	• **National funding sources (15, 23.8%)** - Government (12, 19%) - University/academia (3, 4.8%) • **Regional funding sources (0, 0%)** • **International funding sources (48, 76.27%)** - NGOs (16, 25.3%) - Government (27, 43%) - Health agencies (2, 3.17%) - University/academia (3, 4.8%)
Somalia	13 (0.4%)	5 (0.4%)	• Public academic/university (5, 100.0%)	• **National funding sources (0, 0%)** • **Regional funding sources (0, 0%)** • **International funding sources (8, 100%)** - NGOs (2, 25%) - Health agencies (4, 50%) - University/academia (2, 25%)
Sudan	58 (1.7%)	25 (1.9%)	• Government (5, 20%) • Healthcare organizations (2, 8%) • Intergovernmental (WHO, UN, World Bank; 1, 4%) • Not-for-profit organization (2, 8%) • Private academic/university (8, 32%) • Public academic/university (7, 28%)	• **National funding sources (6, 19.4%)** - NGOs (1, 3.2%) - Government (4, 13%) - University/academia (1, 3.2%) • **Regional funding sources (0, 0%)** • **International funding sources (25, 80.6%)** - NGOs (11, 35.4%) - Government (10, 32.2%) - University/academia (4, 13%)
Syria	18 (0.5%)	7 (0.5%)	• Private academic/university (3, 42.9%) • Public academic/university (4, 57.1%)	• **National funding sources (0, 0%)** • **Regional funding sources (0, 0%)** • **International funding sources (10, 100%)** - NGOs (6, 60%) - Government (3, 30%) - University/academia (1, 10%)
Yemen	18 (0.5%)	6 (0.4%)	• Public academic/university (6, 100%)	• **National funding sources (0, 0%)** • **Regional funding sources (0, 0%)** • **International funding sources (9, 100%)** - NGOs (6, 75%) - Government (3, 25%)
**More than one country**				
More than one “beyond” the region	171 (5%)	101 (7.5%)	• Government (2, 2%) • Healthcare organizations (3, 3%) • Intergovernmental (WHO, UN, World Bank; 4, 4%) • Not-for-profit organization (9, 8.9%) • Private academic/university (20, 19.8%) • Public academic/university (63, 62.4%)	• **National funding sources (3, 2.2%)** - University/academia (3, 2.2%) • **Regional funding sources (17, 12.38%)** - NGOs (4, 2.9%) - Government (1, 0.72%) - Health agencies (3, 2.2%) - University/academia (9, 6.56%) • **International funding sources (117, 85.4%)** - NGOs (34, 25%) - Government (59, 43%) - Health agencies (3, 2.2%) - University/academia (16, 11.6%) - Pharmaceutical industry (5, 3.6%) - Others (2, 1.45%)
More than one within the region	173 (5.1%)	61 (4.5%)	• Government (1, 1.6%) • Healthcare organizations (6, 9.8%) • Intergovernmental (WHO, UN, World Bank; 8, 13.1%) • Not-for-profit organization (2, 3.3%) • Private academic/university (16, 26.2%) • Public academic/university (28, 45.9%)	• **National funding sources (4, 4.7%)** - NGOs (1, 1.2%) - University/academia (3, 3.5%) • **Regional funding sources (7, 8.3%)** - Government (1, 1.2%) - Health agencies (1, 1.2%) - University/academia (4, 4.7%) - Pharmaceutical industry (1, 1.2%) • **International funding sources (74, 87%)** - NGOs (33, 38.8%) - Government (23, 27%) - Health agencies (1, 1.2%) - University/academia (5, 5.9) - Pharmaceutical industry (12, 14.1%)

UN, United Nations, NGO, non-governmental organization

^*^The denominator is 1632 instead of 1635 because two of the funded studies have unclear funding sources and, thus, are not included here

^**^Both Jordan and Iran were previously classified as “upper-middle-income countries” according to the World Bank’s country classifications by income level for 2021–2022 (which coincided with the data collection period of the study); however, they are now classified as “lower-middle-income” countries

### Associations between variables of interest and sources of funding for HPSR articles

There was significant association between country income level and source of funding (*P* < 0.001; Table 3). Countries in the high-income and upper-middle income groups were significantly more likely to be funded by national sources (78.7% versus 77.1%, respectively), more specifically universities, while countries in the low-income groups were significantly more likely to be funded by international sources (87.2%).

**Table 3:** Association between funding source and income levels

	Country classification					
	High income	Low income	Lower middle income	Upper middle income	More than one	*P*-value
**National funding sources**	214 (78.7%)	12 (11%)	224 (49.4%)	363 (77.1%)	3 (7.3%)	< 0.001
NGOs	10 (3.70%)	1 (0.90%)	9 (2%)	5 (1.10%)	0 (0%)	
Government	35 (12.90%)	8 (7.30%)	27 (6%)	37 (7.90%)	0 (0%)	
Health agencies	16 (5.90%)	0 (0%)	7 (1.50%)	3 (0.60%)	0 (0%)	
University/academia	149 (54.80%)	3 (2.80%)	179 (39.50%)	317 (67.30%)	3 (7.30%)	
Pharmaceutical industry	4 (1.50%)	0 (0%)	2 (0.40%)	1 (0.20%)	0 (0%)	
**Regional funding sources**	14 (5.1%)	1 (0.9%)	13 (2.9%)	11 (2.3%)	2 (4.9%)	
NGOs	3 (1.10%)	0 (0%)	2 (0.40%)	2 (0.40%)	0 (0%)	
Government	1 (0.40%)	0 (0%)	2 (0.40%)	2 (0.40%)	0 (0%)	
Health agencies	1 (0.40%)	0 (0%)	1 (0.20%)	2 (0.40%)	0 (0%)	
University/academia	8 (2.90%)	1 (0.90%)	8 (1.80%)	5 (1.10%)	2 (4.90%)	
Pharmaceutical Industry	1 (0.40%)	0 (0%)	0 (0%)	0 (0%)	0 (0%)	
**International funding sources**	44 (16.2%)	95 (87.2%)	216 (47.7%)	97 (20.6%)	35 (85.4%)	
NGOs	15 (5.50%)	39 (35.80%)	75 (16.60%)	49 (10.40%)	16 (39%)	
Government	11 (4%)	41 (37.60%)	94 (20.80%)	33 (7%)	10 (24.40%)	
Health agencies	1 (0.40%)	3 (2.80%)	11 (2.40%)	3 (0.60%)	1 (2.40%)	
University/academia	4 (1.50%)	11 (10.10%)	27 (6%)	8 (1.70%)	5 (12.20%)	
Pharmaceutical industry	13 (4.80%)	1 (0.90%)	9 (2%)	4 (0.80%)	3 (7.30%)	
**Not clear**	0 (0%)	1 (0.90%)	0 (0%)	0 (0%)	1 (2.40%)	

Studies that included at least one country beyond the EMR (for example, as part of international research consortia) were significantly more likely to be funded by international sources (80.2%), while studies focusing on one or more country within the EMR only were significantly more likely to be funded by national sources (65.3%), mainly universities (52%; Table 4).

**Table 4:** Association between funding source and country of focus of HPSR article

	Focus of HPSR article on one or more EMR countries	Focus of HPSR article on at least one country beyond EMR countries in addition to EMR	*P*-value
**National funding sources**	813 (65.3%)	3 (3%)	< 0.001
NGOs	25 (2%)	0 (0%)	
Government	107 (8.6%)	0 (0%)	
Health agencies	26 (2.1%)	0 (0%)	
University/academia	648 (52%)	3 (3%)	
Pharmaceutical industry	7(0.6%)	0 (0%)	
**Regional funding sources**	26 (2.1%)	15 (14.9%)	
NGOs	3 (0.2%)	4 (4%)	
Government	4 (0.3%)	1 (1%)	
Health agencies	3 (0.2%)	1 (1%)	
University/academia	15 (1.2%)	9 (8.9%)	
Pharmaceutical industry	1 (0.1%)	0 (0%)	
**International funding sources**	406 (32.6%)	81 (80.2%)	
NGOs	171 (13.7%)	23 (22.8%)	
Government	148 (11.9%)	41 (40.6%)	
Health agencies	17 (1.4%)	2 (2%)	
University/academia	45 (3.6%)	10 (9.9%)	
Pharmaceutical industry	25 (2%)	5 (5%)	
**Not clear**	0 (0%)	2 (2%)	

Regarding the first author’s country of affiliation, articles with first authors from the EMR were more likely to be funded by national sources (73.9%), mainly universities (61.7%) while articles were the first authors were from non-EMR countries were more likely to be funded by international sources (79.8%) These differences are statistically significant, with a *P*-value < 0.001 (Table 5).

**Table 5:** Association between funding source and the first author’s country of affiliation

	First author’s affiliation from EMR country	First Authors’ affiliation from non-EMR country	*P*-value
**National funding sources**	764 (73.9%)	52 (16.7%)	< 0.001
NGOs	20 (1.90%)	5 (1.60%)	
Government	79 (7.60%)	28 (9%)	
Health agencies	22 (2.1%)	4 (1.3%)	
University/academia	638 (61.7%)	13 (4.2%)	
Pharmaceutical industry	5 (0.5%)	2 (0.6%)	
**Regional funding sources**	30 (2.9%)	11 (3.5%)	
NGOs	3 (0.3%)	4 (1.3%)	
Government	3 (0.3%)	2 (0.6%)	
Health agencies	4 (0.4%)	0 (0%)	
University/academia	20 (1.9%)	4 (1.3%)	
Pharmaceutical industry	0 (0%)	1 (0.3%)	
**International funding sources**	238 (23%)	249 (79.8%)	
NGOs	116 (11.2%)	78 (25%)	
Government	79 (7.6%)	110 (35.3%)	
Health agencies	8 (0.8%)	11 (3.5%)	
University/academia	19 (1.8%)	36 (11.5%)	
Pharmaceutical industry	16 (1.5%)	14 (4.5%)	
**Not clear**	2 (0.2%)	0 (0%)	

Similar funding patterns were observed for COVID-19-related HPSR articles (compared to non-COVID-19-related HPSR articles), with national sources, mainly universities, comprising the main source of funding (78.95% versus 59%). In contrast, international funding sources decreased for COVID-19-related HPSR articles (15.8% versus 37.7%). This difference was statistically significant at *P* < 0.001 (Table 6).

**Table 6:** Association between funding source and COVID-19-related articles

	Non-COVID-19-related articles		COVID-19-related articles		*P*-value for detailed categories	*P*-value for main categories
**National funding sources**	741	59%	75	78.95%	0.001	< 0.001
NGOs	24	2%	1	1.05%		
Government	97	8%	10	10.53%		
Health agencies	20	2%	6	6.32%		
University/academia	593	47%	58	61.05%		
Pharmaceutical industry	7	1%	0	0.00%		
**Regional funding sources**	36	3%	5	5%		
NGOs	7	1%	0	0%		
Government	5	0%	0	0%		
Regional health agencies	4	0%	0	0%		
University/academia	19	2%	5	5%		
Pharmaceutical	1	0%	0	0%		
**International funding sources**	472	37.7%	15	15.8%		
NGOs	186	15%	8	8%		
Government	186	15%	3	3%		
Health agencies	19	2%	0	0%		
University/academia	53	4%	2	2%		
Pharmaceutical industry	28	2%	2	2%		
**Not clear**	2	0.2%	0	0%		

NGO, non-governmental organization

## Discussion

### Summary and interpretation of findings

This is the first study to address the reporting of funding and funding sources in published HPSR articles in the EMR. More than half of the included articles originated from only three out of the 22 EMR countries, namely Iran, the KSA and Pakistan. When it comes to funding, approximately 30% of HPSR papers in the EMR did not report any details on study funding. Among the articles that reported being funded, analysis of funding sources across all country income groups revealed that the most common source was national, followed by international and lastly regional sources. Among the national sources, universities accounted for the majority of funding. Further analysis of funding sources by country income group showed that in low-income and lower-middle-income countries (as classified by the World Bank at the time of data collection), all or the majority of funding came from international sources, while in high-income and upper-middle-income countries, national funding sources, mainly universities, were the primary sources of funding. However, exceptions to this trend included Kuwait, Oman and Libya, where government funding took precedence.

The majority of funded papers’ first authors were affiliated with academia/university while a minority were affiliated with government, healthcare organizations or intergovernmental organizations.

When articles conducted in Iran, which accounted for the highest number of included papers (30%), were excluded from the entire analysis (and not only those related to upper-middle-income countries), a different overall funding pattern emerged. In this scenario, international funding sources took precedence (457, 54.7%), followed by national (336, 40.2%) and regional (40, 4.8%) sources. This is not unexpected, given that, in Iran, the main source of funding is national funding (94%), specifically universities (82.80%). Furthermore, international sanctions have reduced the willingness of international scholars to cooperate with Iranians scholars and students, while also making it difficult for Iranian researchers to receive health-related grants from foreign regional or international organizations [21].

We found the following characteristics to be significantly associated with the funding source: income level, focus of HPSR article (that is, EMR or as part of international research consortia) and first-author affiliation. The latter may be partially explained by the limited expertise in the EMR to generate solid proposals to compete for these grants, restricting HPSR’s access to worldwide competitive funding options [11].

While the COVID-19 pandemic had drastic consequences on health systems, highlighting the importance of HPSR for evidence-informed decision-making, our findings suggest that funding from government did not increase for COVID-19-related HPSR, while universities/academia took the lead in funding COVID-19-related HPSR articles in the EMR. In contrast, international funding for HPSR in the EMR decreased during the pandemic. According to Becerra-Posada et al., given a higher need for funds for medical care and vaccines for COVID-19, research funding may be a lesser priority in countries suffering budgetary restrictions [22]. Thus, the international funding may have been diverted from HPSR to health systems reforms, testing centres, and more health-related and clinical research. 

Some potential limitations of the study are worth noting. Firstly, despite our attempts to enhance the comprehensiveness of our search by utilizing several databases, including IMEMR, which is specific to the EMR, it should be acknowledged that some researchers in the region may publish their research papers on websites or journals not indexed in the databases we searched or these publications might not be available online. We attempted to partially overcome this by searching Google Scholar and screening the reference lists of included as well as relevant articles. Furthermore, our search date for articles extended up to 2022; hence, it does not encompass studies that could have been published after this date. Nevertheless, it is unlikely that such studies would change the findings in a significant way. At the same time, we believe the 12-year review we conducted provides a good analysis of the funding landscape for HPSR in the EMR. Secondly, for articles encompassing multiple countries, we categorized them into two groups: “More than one beyond the region” and “More than one within the region”, maintaining a distinction from individual country analyses. While this categorization may overlook specific contributions from individual countries within these broader categories, the lack of specificity regarding the countries involved made it challenging to merge them accurately with respective national totals. Indeed, several articles referenced broader regions such as “EMR” or “Middle East” without specifying the countries included, potentially leading to inaccuracies if merged without clear delineation. Also, it is worth noting that articles focusing on more than one country contributed to only 10% of the total included articles, suggesting limited implications for our findings. Finally, the income classification for two countries – Iran and Jordan – changed since the completion of the data collection period for this study. However, this is not expected to significantly alter the results, as both countries are still considered middle-income countries, with variations between upper-middle and lower-middle income levels.

### Comparison to other studies and trends

Iran’s leading position in HPSR production has been re-iterated in previous publications [11]. When it comes to reporting of funding for HPSR papers, our findings align with a cross-sectional survey of 400 HPSR studies (200 systematic reviews and 200 primary studies), which revealed that a third of sampled HPSR papers did not provide any information about funding [19]. This is in contrast to clinical papers whereby 89% of clinical trial reports published in 2015 included funding statements [23]. This is a reflection of both a suboptimal compliance by authors with the funding policies and a deficient enforcement by the journals [19].

As for the sources of funding for published HPSR papers, our findings align with reported funding sources for HPSR in LMICs, indicating notably low government spending on HPSR, whereby governments tend to give more consideration to basic science and clinical research over HPSR [24]. In these settings, funding for HPSR primarily originates from international and multilateral aid as well as contracts with larger research consortia, with limited contributions from national governments [25–27]. This funding pattern was particularly evident in low-income and lower-middle income countries of the EMR. Additionally, international funding sources predominated when Iran was excluded from the analysis.

Existing research indicates that the strong dependence of countries on international funding generally comes at the expense of addressing community needs and health system priorities where research topics dictated by funders and donors are prioritized [14, 28, 29]. This concern is further reinforced by another study on HPSR funding in LMIC, which revealed that these countries depend on a narrow array of donors, which puts them at risk of losing funding if the donors’ priorities shift away from HPSR [14]. In addition to that, the reliance on international funding impedes the national authorities from developing local, sustainable capacity to perform HPSR [25].

A number of factors have been identified as influencing investment in or funding for health research in general and HPSR in particular in the EMR. Health research in this region is fragmented and insufficient because of the absence of national policies and strategic plans that promote investment in health systems research [11, 28]. Moreover, in the conflict-affected countries of the region, health systems research is not a top priority of the national and international investments and initiatives [28]. Bureaucratic bottlenecks such as corruption and lack of accountability and unstable government regimes further hamper the establishment and improvement of domestic HPSR funding [29]. Additionally, the weak institutional and infrastructural capacity and the absence of a critical mass, that is, an abundant number of qualified researchers with a uniquely varied skill mix in research institutions hinders HPSR national as well as international funding [8, 11, 25, 27].

### Implications for policy and practice

Study findings indicate limited interest and commitment of governments to HPSR funding in the EMR. We provide key implications for policy and practice moving forward. First and foremost, governments are urged to outline a national vision with clearly defined goals, objectives, policies and strategies for HPSR [30, 31]. The WHO Global Ministerial Forum suggests institutionalizing HPSR and forming a separate institute or department for HPSR, whether as part of ministry of health or not [29, 32]. This would allow for better governance of research, improved management for resources and consequently enhanced credibility and integrity [29, 32]. Also, these institutions and departments can hold national health programs and work on integrating them with those of the external donors [25, 29].

Second, increasing domestic funding for HPSR is needed to reduce reliance on external donors while improving HPSR’s focus on national priorities. To this end, governments in the EMR should establish explicit national funding or a budget line item for HPSR with sustainable and transparent processes in place for mobilizing and allocating funds for HPSR [11]. Additional strategies for increasing domestic funding of HPSR include the formation of advocacy coalitions and continuous advocacy to both public- and private-sector stakeholders. Moreover, the engagement of local stakeholders in research priority-setting exercises, in organizational-level capacity-building to improve the use of research evidence, in assessing the gains of previous funding, and in assigning a portion of international funds to local research teams all enhance demand and funding for HPSR at the national level [31, 33]. Given that funding from international sources will continue to play a role in the region, strong governance to ensure coordinated efforts and alignment to country priorities will be key to attaining maximum return on investment [11]. It is also important to ensure that at least some of this external funding is used to strengthen national researcher capacity as well as sensitize decision-makers to the potential of HPSR to inform improved national policy. Initiatives to promote donor alignment and harmonization such as the International Health Partnership are also a promising option for governments to align their HPSR vision with the funder’s interests [25].

Third, given that increases in domestic funding commitments for HPSR are likely to be difficult to achieve without stronger policy-maker demand for HPSR, it would be important for EMR countries to invest in capacity-building and awareness raising for HPSR to improve the prevailing culture for research and evidence-informed decision-making. Individual-level capacity should be complemented by institutional mandates for policy-makers to use research evidence as input into the decision-making process as well as institutional structures and mechanisms to hold decision-makers accountable for their decisions. Furthermore, given that generating appropriate, trustworthy evidence depends on the existence of good research organizations, building and strengthening academic programs (master’s and PHDs) and institutions for HPSR and knowledge translation would enhance the technical capacities of all HPSR stakeholders and improve the integration of research findings into policy-making. Incentive mechanisms to support knowledge translation work and interdisciplinary research can further incentivize researchers to engage in HPSR and evidence-informed policy-making.

Fourth, considerations could be given to establish a regional strategy for HPSR which articulates the vision and goal for HPSR in the EMR as well as guides resource mobilization and allocation decisions for HPSR, including priority-setting exercises to shape HPSR research agenda in the region. A regional advocacy coalition can also be considered to raise regional funds for HPSR, which can be allocated in an informed manner to support national HPSR initiatives [5, 12]. Moreover, a regional forum or network can be established for raising awareness, building capacity and creating demand for HPSR.

Finally, given that our study identified suboptimal reporting of funding information in HPSR papers, journals need to better enforce their funding policies.

## Conclusion

This is the first study to address the reporting of funding and funding sources in HPSR articles in the EMR. Despite the majority of journals publishing on HPSR requiring the reporting of funding, approximately 30% of HPSR papers did not report on the funding source. Moreover, study findings revealed heavy reliance on universities and international funding sources in funding HPSR articles in the EMR, with a minimal role of national governments and regional entities. Study findings can guide researchers, policy-makers and funders to strengthen and improve the profile of HPSR funding in the EMR.

### Supplementary Information

Supplementary Material 1. Preferred Reporting Items for Systematic reviews and Meta-Analyses extension for Scoping Reviews (PRISMA-ScR) Checklist.

## Data Availability

All data generated or analysed during this study are included in this published article (and its supplementary information files).
